# The *TGFBI* R555W mutation induces a new granular corneal dystrophy type I phenotype

**Published:** 2011-01-20

**Authors:** Yanan Zhu, Xingchao Shentu, Wei Wang

**Affiliations:** Eye Center of the 2nd Affiliated Hospital, Medical College of Zhejiang University, Hangzhou, China

## Abstract

**Purpose:**

To report the clinical and molecular features of a distinct form of transforming growth factor-β-induced (*TGFBI*) gene-linked corneal dystrophy exhibiting a new granular corneal dystrophy type I (CDGG1) phenotype.

**Methods:**

A complete ophthalmologic examination was performed in all individuals of a Chinese family in which autosomal dominant transmission of the disease had been observed. DNA was obtained from the peripheral blood leukocytes of each participating subject. Genetic analyses included keratin 3 (*KRT3*), keratin 12 (*KRT12*), and *TGFBI* polymerase chain reaction (PCR) amplification and automated nucleotide sequencing of exons from the genomic DNA.

**Results:**

The corneal phenotype in this pedigree was characterized by multiple bilateral dot-like, circular opacities at different corneal depths, with some of the affected individuals only having opacities in the epithelium, which is different from the typical CDGG1 phenotype. *TGFBI* analysis revealed a heterozygous point mutation at exon 12 (c.1663C>T) in all of the affected individuals, predicting a p.R555W missense mutation.

**Conclusions:**

The phenotype which resulted from the *TGFBI* R555W mutation in this family is distinct from that observed in the typical case of CDGG1. We propose this disorder should be classified as a new phenotype of CDGG1, and this finding demonstrates the importance of gene diagnosis in the corneal dystrophies.

## Introduction

The corneal dystrophies (CDs) are genetically determined diseases in which the progressive opacification of the corneal layers results in a variable loss of transparency and eventually in visual impairment [1]. Clinically, the corneal dystrophies can be divided into three groups based on the sole or predominant anatomic location of the abnormalities. Some affect primarily the corneal epithelium and its basement membrane or Bowman layer and the superficial corneal stroma (anterior corneal dystrophies), the corneal stroma (stromal corneal dystrophies), or the Descemet's membrane and the corneal endothelium (posterior corneal dystrophies) [2].

Sometime it is difficult to the clinical classification of a special case. Since overlapping mutations can induce very similar phenotypes, it is easy to appreciate the difficulty in trying to distinguish them clinically. Therefore, molecular genetic studies have come to have an important role in the diagnosis of CDs, and the majority of CDs recognized to date are transmitted in an autosomal dominant mode of transmission with a high degree of penetrance [3]. An apparent genotype–phenotype correlation has emerged from these molecular studies, as four distinct heterozygous recurrent mutations in the transforming growth factor-β-induced gene (*TGFBI*) are associated with four specific phenotypes: p.R555W in granular corneal dystrophy type I (CDGG1), p.R124C in Lattice CD type I, p.R124H in granular CD (GCD) type II, and p.R555Q in Thiel-Behnke CD [3-5].

Corneal granular dystrophies are a group of disorders which were originally described by Groenouw [6] and are some of the most common corneal dystrophies seen in clinical practice [7]. The most typical kind of corneal granular dystrophy, CDGG1 (OMIM 121900), is characterized by small, discrete, sharply demarcated grayish white opacities resembling bread crumbs or snowflakes in the anterior central stroma. The histological findings are typically hyaline degeneration and absence of acid mucopoly-saccharide depositions.

This atypical corneal dystrophy in a Chinese six-generation family which was identified using molecular genetic studies, is proposed as a new phenotype of CDGG1, and confirms the relationship between CDGG1 and p.R555W in the *TGFBI* gene.

## Methods

### Family data and genomic DNA preparation

**Figure 1:**
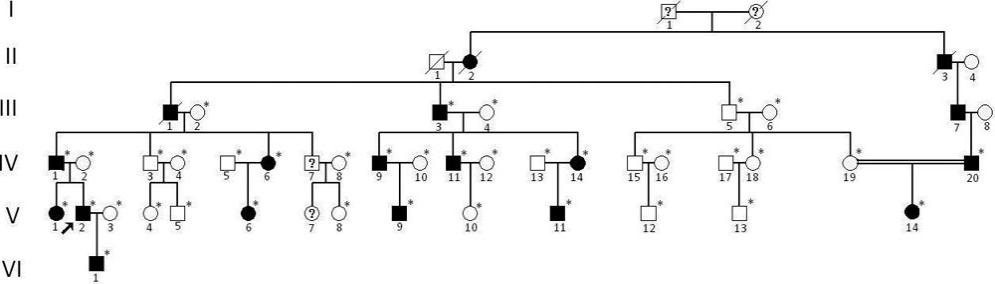
Pedigree demonstrating the six generations of affected members in a Chinese family having a p.R555W mutation in the *TGFBI* gene. The filled symbols represent the affected individuals. *=Individuals in whom molecular genetic investigation was performed. The arrow indicates the proband.

A six-generation family with atypical corneal dystrophy was identified through the Eye Center of the 2nd Affiliated Hospital, Medical College of Zhejiang University, Hangzhou, China. Appropriate informed consent was obtained from all participants and the study protocol followed the principles of the Declaration of Helsinki. Thirty-eight individuals (14 affected and 24 unaffected) from the family took part in the study (Figure 1). The affected status was determined by ophthalmologic examination, including visual acuity, slit lamp, and fundus examination. The phenotypes were documented by slit lamp photography.

### Genomic DNA preparation

We collected blood specimens (5 ml) in EDTA and extracted genomic DNA in the peripheral blood leukocytes obtained from the available family members using a Simgen Blood DNA mini kit (Simgen, Hangzhou, China).

### Mutation screening

Gene specific PCR primers for keratin 3 (*KRT3*), keratin 12 (*KRT12*), and *TGFBI* were designed which flanked each exon and intron-exon junction (PCR primers for *TGFBI* are shown in Table 1). The PCR cycling conditions were as follows: 95 °C preactivation for 5 min, 10 cycles of touchdown PCR with a 1 °C reduction per cycle from 60 °C to 50 °C, followed by 25 cycles with denaturation at 95 °C for 25 s, annealing at 55 °C for 25 s, and extension at 72 °C for 40 s and a final extension at 72 °C for 10 min. PCR products were isolated by electrophoresis on 1.5% agarose gels and sequenced using the BigDye Terminator Cycle sequencing kit V 3.1 (ABI Applied Biosystems; Genscript Co., Nanjing, China) on an ABI PRISM 3730 Sequence Analyzer (ABI), according to the manufacturer’s instructions.

**Table 1:** Primers used in polymerase chain reaction of the *TGFBI* gene.

**Exon**	**Primers**	**Sequence**	**Product size**	**Reference**
1	1F	GCGCTCTCACTTCCCTGGAG	247 bp	[8]
	1R	GACTACCTGACCTTCCGCAG		[8]
2	2F	GAGTCATTAAAGTGGGGTGGA	253 bp	[8]
	2R	CAACCTGACCTTCTAGAAGGCT		[8]
3	3F	ACTTAGTGGAGAGGGGCCAGA	206 bp	[9]
	3R	TCCTCTCTCCCACCATTCCCTT		[9]
4	4F	TCAGAGAAGGGAGGGTGTGGTT	292 bp	[10]
	4R	CCTCGGGGAAGTAAGGCAGTT		[10]
5	5F	CAGAGTTGCAAGGACCCATCT	308 bp	[10]
	5R	AGCCCACACATGGAACAGAAATG		[9]
6	6F	ACACTGTTTTCTCCTCCCGG	299 bp	[10]
	6R	AGAGTTCCTGCTAGGCCCCTCTT		[9]
7	7F	TGTGCATCTTCTGTGGGGAGT	233 bp	[10]
	7R	AGAGCAGGTCTTGGGCTCAA		[10]
8	8F	TGCAAGTGGTCCCTGAGGTTAT	343 bp	[11]
	8R	CACAAAGGATGGCAGAAGAGA		[12]
9	9F	CCCTGGGGTGGATGAATGATAAA	285 bp	[9]
	9R	AGAGTGTTGTGGAGGTGACAG		[10]
10	10F	TCACTTGGTTTCTCAATCCCTG	376 bp	This study
	10R	CTCATGCAAACTCCTGCTTATTG		This study
11	11F	ACCCTGCTACATGCTCTGAAC	282 bp	[10]
	11R	CACATCCCACTCCAGCATGAC		[10]
12	12F	TCTCAGCGTGGTGAGGTATT	257 bp	[10]
	12R	CCCTGAGGGATCACTACTTT		[13]
13	13F	CCTCCTTGACCAGGCTAATTAC	255 bp	[13]
	13R	TGAGATATGTCCTGGAGCCCT		[11]
14	14F	CTGTTCAGTAAACACTTGCTG	257 bp	[13]
	14R	CCACCAACTGCCACATGGAGAA		This study
15	15F	CTCAGTCACGGTTGTTATGA	218 bp	[12]
	15R	TCTATGGCCCAAACAGAGGA		This study
16	16F	CATTGTCATAAGCAGTTGCAG	174 bp	[12]
	16R	ATACAGCAGATGGCAGGCTT		[10]
17	17F	TCCTAGACAGACATGGGGAGAT	426 bp	This study
	17R	TGAGAGAAATTGGCGGAGAG		This study

## Results

### Clinical evaluation

**Figure 2:**
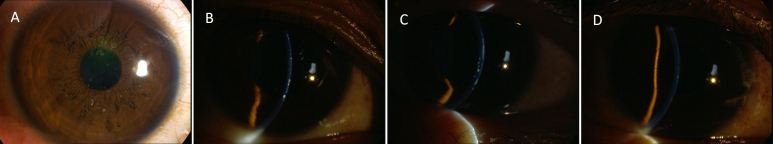
Clinical photographs of patients with atypical CDGG1. **A**: A slit lamp photograph of the proband's cornea (Figure 1, V2) showing multiple round and dot-like grayish white opacities in the central stroma. **B**: A slit lamp photograph of the proband's cornea showing the opacities in the epithelium and anterior stroma. **C**: A slit lamp photograph of the cornea in patient IV6 (Figure 1, IV6) showing the opacities in the epithelium and total stroma. **D**: A slit lamp photograph of the cornea in patient IV20 (Figure 1, IV20) showing opacities only in the epithelium.

The six-generation Chinese family displayed the hallmarks of autosomal dominant inheritance (Figure 1). To confirm the clinical diagnosis, all individuals from whom DNA had been obtained underwent slit lamp examination. The characteristic lesions in the cornea are dot-like or round, discrete, sharply demarcated grayish white opacities, and are found mainly in the central region. The appearance of the lesions was almost the same as typical CDGG1, but of the lesion depths were distinctly different. Furthermore, the lesion depths were found to be different between the affected individuals (Figure 2).The proband and another five members had corneal opacities in epithelium and anterior stroma bilaterally, but four out of fourteen members had opacities only in the epithelium, while four other members had opacities deeper than the middle stroma (Table 2). Most of the affected individuals noticed visual impairment after approximately the age of ten, and then their visual acuity decreased gradually with occasional photophobia. There was no family history of other ocular or systemic abnormalities aside from age-related disorders.

**Table 2:** Depth of cornea lesions in affected individuals.

**Depth of cornea lesions**	**Patient's number (age)**
Epithelium	IV11 (9Y), VI1 (7Y), IV20 (40Y), V14 (10Y)
Epithelium and anterior stroma	III3 (70Y), IV9 (41Y), IV14 (34Y), V1 (29Y), V2 (28Y), V6 (23Y)
Epithelium, anterior and middle stroma	IV1 (52Y), V9 (15Y)
Epithelium and total stroma	IV6(46Y), IV11(41Y)

### Mutation screening

**Figure 3:**
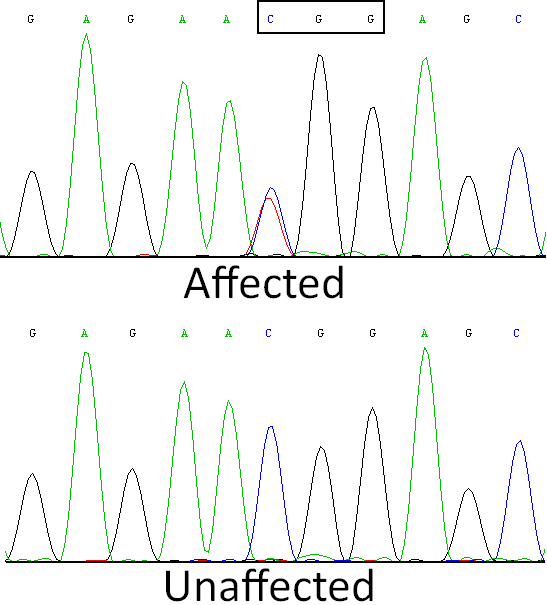
Sequence chromatogram from exon 12 of the *TGFBI* gene showing the region around codon 555 to illustrate a heterozygous C to T transition in the affected individuals. The sense strand is shown; codon 555 is boxed.

Bidirectional sequencing of the coding regions of the candidate genes revealed the heterozygous change of C>T at position 1663 (c.1663 C>T) of *TGFBI* in each of the three affected individuals. This substitution was not seen in the unaffected members or in 100 unrelated control subjects from the same Chinese population, as tested by bidirectional sequence analysis (Figure 3). This substitution results in the replacement of a highly conserved Arginine by Tryptophan at amino acid position 555 (p.R555W) in the fourth internal domain of TGFBIp (The protein encoded by *TGFBI*).

## Discussion

The proband reported here had grayish-whie dot-like circular opacities with some erosive lesions in the epithelium and only a few opacities in the anterior stroma. Therefore, the first clinical impression is of a kind of anterior CD primarily affecting the corneal epithelium and its basement membrane or Bowman layer and the superficial corneal stroma. The mutations reported in the cases of the anterior CDs include the keratin genes *KRT3* on chromosome 12q13 and *KRT12* on chromosome 17q12 [8-10], and the *TGFBI* gene on chromosome 5q31, which is linked to both Reis-Bücklers and Thiel-Behnke dystrophies [5,7]. Thiel–Behnke is also linked to a mutation on chromosome 10q23–24, although the gene product is currently unknown [11].

We use the functional candidate gene analysis approach, and sequenced the PCR product of the candidate genes *KRT3*, *KRT12*, and *TGFBI*. Instead of finding the mutation site previously reported to be related to the anterior CDs, we found a mutation site (C.1663C>T) in *TGFBI* causing a protein change (p.R555W) which has been reported to have a definite relationship with CDGG1 [3-5].

It was found that out of the 14 affected members, 4 had opacities only in the epithelium, 6 had opacities mainly in the epithelium, and a few in the anterior stroma (just like the proband), 2 had opacities in the epithelium and the anterior and middle stroma, and 2 had opacities in the epithelium and total stroma equivalent to the typical CDGG1 phenotype. Most of the affected individuals noticed visual impairment shortly after the age of ten, and then their visual acuity decreased gradually with occasional photophobia. The decrease in visual acuity in the affected members were related with increases of irregular astigmatism and was presumed to be related to the opacities in the pupil area of cornea. All of the affected family members have distance visual acuity better than 20/80 without any need of corneal transplant, so it was not possible to perform histological analysis.

A precise diagnosis of corneal disorders cannot rely solely on the clinical or histological features of the pathology. Certain mutations in *TGFBI* appear to be associated with specific phenotypes of corneal disease regardless of ethnic group. Since the c.1663C>T (p.R555W) mutation in *TGFBI* was first identified for CDGG1 by Munier et al. [12], this mutation has been shown to have a clear genotype-phenotype correlation with CDGG1 [3-5], and CDGG1 in association with the p. R555W mutation is reported to be the most prevalent type of GCD in several different ethnic groups [13-16]. Therefore, genetic investigation is useful in the diagnosis of CDGG1, especially the atypical cases. In this study, we provide clinical and molecular evidence supporting the occurrence of an atypical CDGG1 phenotype attributable to the TGFBI p.R555W mutation.

The typical CDGG1 is characterized by grayish-white granules in the anterior central stroma, and as the disease advances, the number and extent of the opacities increase and the epithelium is progressively involved. In the Classification of Corneal Dystrophies III (IC3D), CDGG1 is described in children as a corneal disease with a vortex pattern of brownish granules superficial to Bowman's layer but not to the epithelium, and in later life, granules may extend into the deeper stroma down to Descemet's membrane [17]. In contrast with the former phenotype, we propose this Chinese CDGG1 case as a new phenotype which is characterized at first by first grayish-white granules in the corneal epithelium and subsequently by progression into the deeper stroma. The rate of progression was different among the family members. For most of the affected members, corneal lesions had developed in the stroma after after the age of approximately ten, but one 40-year-old man still only has opacities in the epithelium, indicating phenotype heterogeneity in this family. Environmental factors may contribute to this interfamily variability in phenotypic expression. As some patients still have as yet only mild clinical manifestations, regular clinical follow-up will be performed.

The protein encoded by *TGFBI* is βig-h3 (TGFBIp, Keratoepithelin), which consists of an NH_2_-terminal Cys-rich EMI domain and four consecutive fasciclin 1 (FAS1) domains [18]. The four FAS1 domains in human TGFBIp correspond to amino acids 134–236 (FAS1–1), 242–372 (FAS1–2), 373–501 (FAS1–3), and 502–632 (FAS1–4). The p.R555W mutation is located in the FAS1–4 region, and is predicted to alter either protein solubility or stability rather than protein structure [19]. Runager et al. [20] suggested a replacement by a hydrophobic amino acid residue (Trp) rather than a positively charged hydrophilic (Arg) in this position may be likely to increase the propensity of the protein to associate, potentially lowering the solubility, simply by reducing the level of electrostatic repulsion. An increased level of intact R555W TGFBIp has been observed in corneal deposits in vivo, supporting that the mutant protein is resistant to degradation and that an increase in protein stability may indeed have pathological consequences [21]. Moreover, Morand et al. [22] reported that the mutation p.R555W in TGFBIp triggered apoptosis in human corneal epithelial cells, and suggested that aberrant activation of the α3β1 integrin-related pathway by mutated TGFBIp is part of the pathophysiological process that leads to the TGFBI-related CDs.

Previous studies which conducted immunohistochemical and biochemical analyses on the nature of the abnormal deposits in the CDs revealed the TGFBIp to be a major component in all types of *TGFBI*-related CD [14,21,23]. The deposits have been precisely localized in the stroma for CDGG1 [5,18,24]. Based on our case, we suggest that the deposits in CDGG1 can also first appear in the epithelium.

In summary, we report a Chinese CDGG1 pedigree with an atypical phenotype characterized corneal lesions first appearing in the epithelium. This finding expands the spectrum of the CDGG1 phenotypes caused by the p.R555W mutation in the *TGFBI* gene, and demonstrates the importance of gene diagnosis in the corneal dystrophies.
